# Association of lymph vessel density with occult lymph node metastasis and prognosis in oral squamous cell carcinoma

**DOI:** 10.1186/s12903-021-01459-6

**Published:** 2021-03-11

**Authors:** Simone E. S. Faustino, Kellen C. Tjioe, Agnes Assao, Michele C. Pereira, André L. Carvalho, Luiz P. Kowalski, Denise T. Oliveira

**Affiliations:** 1 Department of Surgery, Stomatology, Pathology, and Radiology, Area of Pathology, Bauru School of Dentistry, University of São Paulo, Alameda Octávio Pinheiro Brisolla, 9-75, Bauru, São Paulo 17012-901 Brazil; 2 Oral Oncology Center, Aracatuba School of Dentistry, São Paulo State University (Unesp), Aracatuba, São Paulo Brazil; 3 Federal University of São João Del Rei – Midwest Campus Dona Lindu, Divinópolis, Minas Gerais Brazil; 4 Department of Head and Neck Surgery, Barretos Cancer Hospital, Barretos, São Paulo Brazil; 5 Department of Head and Neck Surgery and Otorhinolaryngology, A.C. Camargo Hospital, São Paulo, São Paulo Brazil

**Keywords:** Oral squamous cell carcinoma, Lymphatic metastasis, Prognosis, Podoplanin, D2-40

## Abstract

**Background:**

The aims of this study were to determine intra (ILVD) and peritumoral (PLVD) lymphatic vessel density (LVD), and to investigate the relationship of LVD with occult metastasis and prognosis.

**Methods:**

Eighty-seven oral squamous cell carcinomas, in clinical stages I or II, arising in the tongue or floor of the mouth were stained with podoplanin. Lymphatic vessels were quantified in intra and peritumoral areas by sequential analysis and hot spot evaluation. Associations of the ILVD and PLVD with clinicopathologic parameters were determined by Chi-square or Fisher’s exact test. The 5 and 10-year survival rates were calculated by the Kaplan–Meier and compared using the log-rank test.

**Results:**

No significant association was observed between ILVD or PLDV and clinicopathologic variables including occult lymph node metastasis, or clinical follow-up. However, ILVD showed a significant association with regional recurrence (p = 0.040). The perineural invasion was associated with PLVD (p = 0.041). Disease-specific (p = 0.044) and disease-free survivals (p = 0.016) had significant association with PLVD.

**Conclusions:**

The intra or peritumoral lymphatic vessel density had no predictive value for occult lymph node metastasis in the early stages of oral cancer arising in the tongue or floor of mouth.

**Supplementary Information:**

The online version contains supplementary material available at 10.1186/s12903-021-01459-6.

## Introduction

Oral squamous cell carcinoma (OSCC), among other solid malignant tumors, preferentially disseminate through the complex lymphatic system into the regional basin. From this point, cancer cells may be transported into the circulation and spread to distal organs and tissues through blood vessels [1, 2]. The ability of the tumoral cells to transpose the lymphatic wall is considered one of the earliest events in metastatic disease [3, 4]. Notably, the occurrence of metastasis is a critical event with severe negative impact on the prognosis of the patient [5]. Thus, predicting the metastatic potential of the tumors, especially of those in more initial stages, is a challenge.

The TNM system is the most used one to stage OSCC clinically and to guide the treatment plan owing its proclaimed prognostic value [5]. However, TNM system takes into account only clinical aspects of the tumor and does not consider its molecular characteristics [6]. Considering the heterogeneity within the tumoral bulk of OSCC and also its diversity among the individuals, there is an understanding that the histologic and molecular features might be evaluated to better recognize the behavior of OSCC and, ultimately, contribute to the prediction of the evolution of the tumor. Furthermore, TNM system has been shown to poorly predict the prognosis of early OSCC [7].

In OSCC, the incidence of occult metastasis in neck lymph nodes after the initial treatment ranges from 23.1 to 45% [8, 9]. These high numbers constitute a strong argument favoring the elective neck dissection in tongue and floor of the mouth OSCC in earlier stages (clinical stages I and II) [9, 10]. On the other hand, the dissection is an invasive procedure that is unnecessary in patients whose neck are negative. Therefore, it is important to identify the prognostic factors that influence the occurrence of regional lymph node metastasis in early-stage OSCC. Knowing which patients are at risk, and instituting proper treatment early in the course of the disease should reduce the incidence of occult metastasis and improve survival rates [9–11].

In OSCC, high lymphatic vessel density (LVD) was found to be associated with cervical lymph node metastasis [12–18], increased risk of local recurrence [11, 18], and lower survival [11, 15, 18]. However, most of the studies includes patients with early as well as late stages of the tumor. Furthermore, recent investigations have renewed the debate, as the LVD has not been associated with the aggressiveness [19, 20] or survival rates of OSCC patients [21]. Another recent data, a systematic review, suggested that lymphatic vessel markers may be a reliable prognosticator for tongue SCC, however the authors highlighted the need of studies with larger patient cohorts [6]. Thus, whether lymphatic vessel density is an important prognostic factor for early stage OSCC has yet to be addressed and motivated this study. Our aims were to analyze the intra (ILVD) and peritumoral (PLVD) lymph vessel density in stage I and II OSCC and to investigate its relationship with occult metastasis in cervical lymph nodes and patient’s prognosis.

## Materials and methods

### Patients and specimens

This study was based on the analysis of 87 patients who underwent surgical treatment for primary OSCC, between 1968 and 2001, at the Head and Neck Surgery and Otorhinolaryngology Department of the A.C. Camargo Cancer Center, São Paulo, Brazil. The study protocol was approved by the institutional ethics committee (protocol number: 746/05) and was performed according to the Helsinki declaration principles. The informed consent was waived by the A.C. Camargo Cancer Center ethics committee once it was a retrospective study that included the analysis of specimens that were collected previously for diagnostic and/or treatment purposes. All patients included in this study were previously analyzed by Faustino et al*.* [22].

The inclusion criteria of the patients were: (i) primary OSCC located in the floor of the mouth or oral tongue, confirmed by biopsy, (ii) clinical stages I (T1N0M0) or II (T2N0M0); (iii) patients without other simultaneous primary tumors; (iv) patients who did not undergo radiotherapy, chemotherapy or other treatment prior to surgery; (v) complete clinical and follow-up data; and (vi) tumor tissue available for microscopic analysis.

Clinical data of the patients were obtained from the medical records and included age, gender, ethnic group, tobacco and alcohol consumption, tumor location, TNM stage [5], treatment (surgery, postoperative adjuvant radiotherapy), and clinical follow-up.

Information on the presence of vascular embolization, as well as perineural, muscular and salivary gland infiltrations, was retrieved from pathology reports. The resection margin status was recorded for each OSCC.

A formalin-fixed 3-µm section of OSCCs was taken from the pathology archive for hematoxylin–eosin (HE) staining analysis. Three previously trained examiners, blinded to the clinical data, analyzed the specimens under a light microscope (Axioskop2 Plus, Zeiss, Oberkochen, Germany). The tumors were classified according to the malignancy grading by Bryne et al*.* [23].

### Immunohistochemistry

Immunoreactivity against podoplanin was assessed using the standard streptavidin–biotin-peroxidase complex method, as previously described [22]. Briefly, the specimens were incubated with the primary monoclonal antibody anti-podoplanin (Novus Biological, Littleton, CO, USA) at 1:200, overnight at 4 °C. Then, podoplanin-stained tumor sections were incubated with the appropriate secondary antibody using the kit StreptABComplex/HRP Duet, Mouse/Rabbit (Dako A/S, ref K0492, Denmark). The staining was revealed using 3.3′diaminobenzidine tetrahydrochloride (cod# D-5637; Sigma-Aldrich, St. Louis, Missouri, USA) and the sections were counterstained with Mayer’s hematoxylin. Human lymphangioma was used as positive control. Normal oral mucosa from the surgical margins was used as the internal control. The primary antibody was omitted during immunohistochemical staining for the negative controls.

### Immunostaining evaluation

**Fig. 1:**
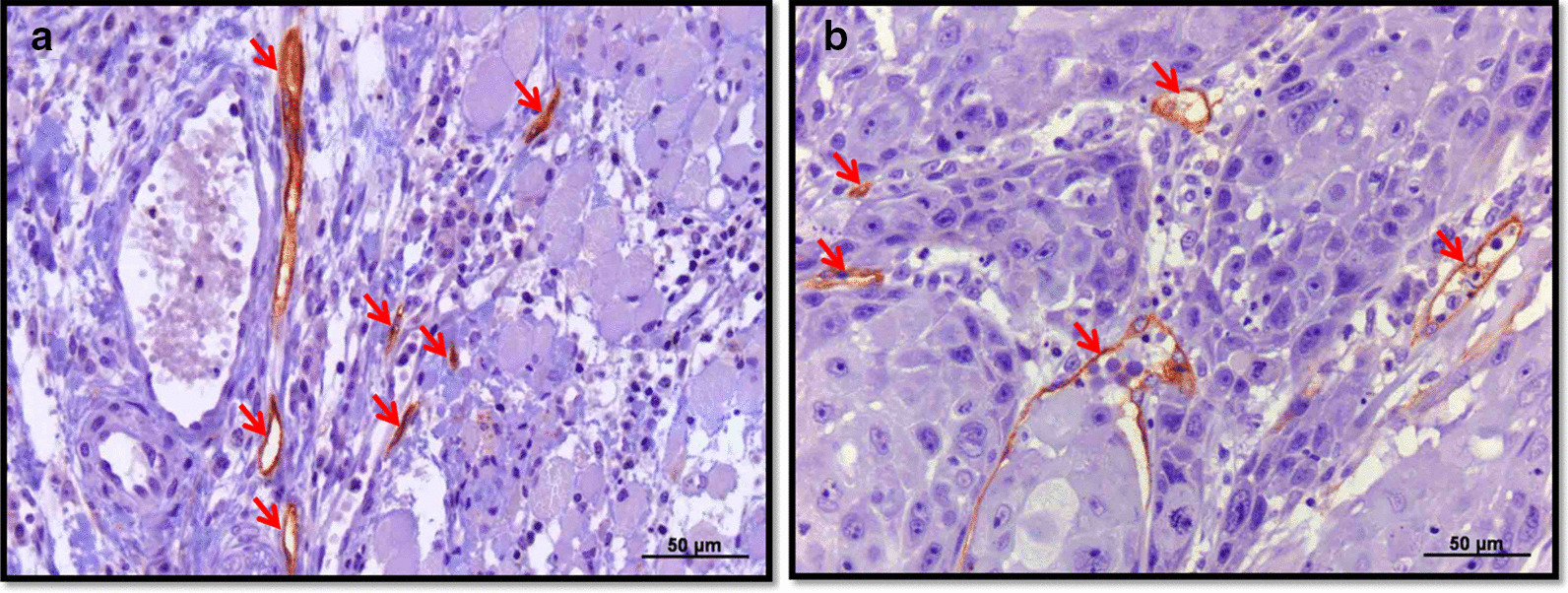
**a** Peri- and **b** intra-tumoral lymphatic vessels in oral squamous cell carcinoma. Lymphatic vessels are indicated with red arrows (IHQ, monoclonal antibody anti-podoplanin, original magnification, 200x)

Podoplanin was used as a selective marker for lymphatic vessels. PLVD (Fig. 1a) and ILVD (Fig. 1b) were determined by two distinct methods in one representative section of each tumor: along the invasive front (sequential) and in the fields with the highest vascular density (hot spot). All lymphatic vessels were counted by two examiners (S.E.S.F. and D.T.O.) simultaneously using the software Axiovision (Axiovision 4.6; Zeiss, Germany).

#### Sequential analysis

As for the sequential analysis, images of the region of interest were digitally captured for further analysis. For PLVD determination, 20 images (× 400 magnification) each tumor were taken in the invasive front. Concerning ILVD, 10 microscopic fields, on average, were selected within the tumor, near the invasive front. Additional 10 microscopic fields from the center of the tumor, avoiding areas of necrosis, were also obtained. An area of approximately 1.88 mm^2^ were analyzed per tumor. PLVD and ILVD were determined by the sum of the total number of lymphatic vessels in all microscopic fields divided by the total area covered.

#### Hot spot evaluation

PLVD and ILVD were evaluated in five tumor fields with highest lymph vascular density (hot spots), using a light microscope at X400 magnification. The total number of lymph vessels obtained in the five fields of each tumor was summed and divided by the total area covered (0.47 mm^2^). The average number of lymphatic vessels per square millimeter was then obtained. Calculation of the arithmetic mean of the LVD by hot spot for all specimens gave PLVD and ILVD.

### Statistical analyses

The statistical analysis was performed using Statistical Package for the Social (version 17.0, SPSS Inc., Chicago, IL, U.S.A.). The associations of PLVD or ILVD with clinical and microscopic variables and occurrence of occult lymph node metastases were verified using the Chi-square or Fisher´s exact test. The 5 and 10-year survival rates (overall and disease-free survivals) were calculated by the Kaplan–Meier method and compared using the log-rank test. *p* values under 0.05 were considered statistically significant.

## Results

### Clinicopathological parameters

Out of the 87 OSCC patients, (78.2%) were males [22]. With regard to ethnic group, 92.0% were Caucasian [22]. Age ranged from 35 to 89 years (mean = 59.4 years). Tobacco or alcohol consumption was documented in 82.8% and 75.9%, respectively. The tongue was the primary site in 69.0% of patients. Based on the International Union Against Cancer (UICC) classification of oral cavity carcinomas, most of the tumors were classified as T2 (67.8%).

All patients underwent primary tumor resection, and 62.3% were also submitted to ipsilateral neck dissection. Nine patients were submitted to bilateral neck dissection. Only 21.8% of the patients had postoperative adjuvant radiotherapy.

Local and regional recurrences occurred in 17.2% and 16.1% of the patients, respectively. Two OSCC patients (2.3%) developed distant metastases and a second primary tumor was detected in 31.0% of the cases.

The majority of OSCC (59.8%) showed no vascular embolization. Perineural (50.6%), muscular (81.6%), and salivary gland (33.3%) infiltrations were also seen. Fourteen patients (16.1%) were lymph node positive, as confirmed by histopathological analysis, at the time of primary tumor resection. Surgical margins were negative in 84 patients (96.6%).

According to the histopathological malignancy grading described by Bryne et al*.* [23], the final scores ranged from 6 to 17 points. Most of the OSCC were well-to-moderately differentiated, characterized by moderate keratinization (31.0%), little nuclear polymorphism (40.2%) and marked inflammatory infiltrate (57.5%), as well as 2–3 mitotic figures per high-power field (52.9%). Forty-four OSCC (50.6%) displayed solid cords of neoplastic cells as the pattern of invasion.

### PLVD

#### Sequential analysis

The mean PLVD was 17.97 lymphatic vessels/mm^2^. For the purpose of statistical analysis, tumors with less than 18 lymphatic vessels/mm^2^ were considered to have low PLVD, whereas those with PLVD greater than or equal to 18 lymphatic vessels/mm^2^ were considered to have high PLVD. There was no statistically significant association between PLVD and demographic and clinicopathologic characteristics or clinical outcome. Furthermore, no association was observed between PLVD and occult lymph-node metastases, or histopathological malignancy grading, as showed in Table 1.

**Table 1:** Association between peritumoral lymphatic vessel density and clinicopathologic variables in oral squamous cell carcinomas. A.C.Camargo Cancer Hospital. São Paulo, Brazil

Variable	Peritumoral lymphatic vessel density					
	Sequential analysis		*p value*	Hot spot evaluation		*p value*
	Low	High		Low	High	
Gender						
Male Female	35 (77.8%) 10 (22.2%)	33 (78.6%) 09 (21.4%)	0.929	32 (76.2%) 10 (23.8%)	36 (80.0%) 09 (20.0%)	0.667
Ethnic group						
White Not white	40 (88.9%) 05 (11.1%)	40 (95.2%) 02 (4.8%)	0.435	37 (88.1%) 05 (11.9%)	43 (95.6%) 02 (4.4%)	0.255
Age						
≤ 59 years > 59 years	22 (48.9%) 23 (51.1%)	23 (54.8%) 19 (45.2%)	0.584	21 (50.0%) 21 (50.0%)	24 (53.3%) 21 (46.7%)	0.756
Tobacco^#^						
No Yes	05 (11.9%) 37 (88.1%)	04 (10.3%) 35 (89.7%)	1.000	05 (13.2%) 33 (86.8%)	04 (9.3%) 39 (90.75)	0.728
Alcohol^#^						
No Yes	09 (21.4%) 33 (78.6%)	06 (15.4%) 33 (84.6%)	0.484	06 (15.8%) 32 (84.2%)	09 (20.9%) 34 (79.1%)	0.552
Tumor site						
Tongue Floor of mouth	30 (66.7%) 15 (33.3%)	30 (71.4%) 12 (28.6%)	0.631	31 (73.8%) 11 (26.2%)	29 (64.4%) 16 (35.6%)	0.345
T stage						
T1 T2	12 (26.7%) 33 (73.3%)	16 (38.1%) 26 (61.9%)	0.254	12 (28.6%) 30 (71.4%)	16 (35.6%) 29 (64.4%)	0.486
Local recurrence						
No Yes	34 (75.6%) 11 (24.4%)	38 (90.5%) 04 (9.5%)	0.066	34 (81.0%) 08 (19.0%)	38 (84.4%) 07 (15.6%)	0.667
Regional recurrence						
No Yes	38 (84.4) 07 (15.6)	35 (83.3%) 07 (16.7%)	0.888	36 (85.7) 06 (14.3%)	37 (82.2%) 08 (17.8%)	0.658
Lymphatic embolization						
No Yes	30 (66.7%) 15 (33.3%)	32 (76.2%) 10 (23.8%)	0.327	28 (66.7%) 14 (33.3%)	34 (75.6%) 11 (24.4%)	0.360
Blood embolization						
No Yes	39 (86.7%) 06 (13.3%)	36 (85.7%) 06 (14.3%)	0.898	37 (88.1%) 05 (11.9%)	38 (84.4%) 07 (15.6%)	0.622
Perineural infiltration						
No Yes	18 (40.0%) 27 (60.0%)	25 (59,5%) 17 (40.5%)	0.069	16 (38.1%) 26 (61.9%)	27 (60.0%) 18 (40.0%)	**0.041**
Muscular infiltration						
No Yes	06 (13.3%) 39 (86.7%)	10 (23.8%) 32 (76.2%)	0.208	05 (11.9%) 37 (88.1%)	11 (24.4%) 34 (75.6%)	0.131
Salivary gland infiltration						
No Yes	25 (55.6%) 20 (44.4%)	33 (78.6%) 09 (21.4%)	0.230	27 (64.3%) 15 (35.7%)	31 (68.9%) 14 (31.1%)	0.649
Lymph-nodal status^#^						
pN0 pN +	30 (81.1%) 07 (18.9%)	20 (74.1%) 07 (25.9%)	0.503	26 (76.5%) 08 (23.5%)	24 (80.0%) 06 (20.0%)	0.733
Malignancy grading						
More differentiated Less differentiated	35 (77.8%) 10 (22.2%)	34 (81.0%) 08 (19.0%)	0.715	32 (76.2%) 10 (23.8%)	37 (82.2%) 08 (17.8%)	0.488

^#^ Excluding patients with lost records

#### Hot spot evaluation

The mean value of PLVD was 37.75 lymphatic vessels/mm^2^. For statistical analyses, OSCCs were classified as having low PLVD (< 38 lymphatic vessels/mm^2^) or high PLVD (≥ 38 lymphatic vessels/mm^2^). There was no association between PLVD and demographic, clinical or patient outcome (Table 1). The presence of perineural invasion was found to be significantly associated with PLVD (p = 0.041, Table 1)*.*

### ILVD

#### Sequential analysis

The mean ILVD was 22.51 lymphatic vessels/mm^2^. OSCCs were classified as having low ILVD (< 23 vessels/mm^2^) or high ILVD (≥ 23 vessels/mm^2^). No significant association was seen between ILVD and demographic or clinicopathologic features, nor histopathologic classification or clinical follow-up (Table 2). Although eight out of 14 patients with occult lymph-node metastasis had high ILVD, no statistical association was observed (*p* = 0.155).

**Table 2:** Association between intratumoral lymphatic vessel density and clinicopathologic variables in oral squamous cell carcinomas. A.C.Camargo Cancer Hospital. São Paulo, Brazil

Variable	Intratumoral lymphatic vessel density					
	Sequential analysis		*p value*	Hot spot evaluation		*p value*
	Low	High		Low	High	
Gender						
Male Female	43 (86.0%) 07 (14.0%)	25 (67.6%) 12 (32.4%)	**0.040**	38 (82.6%) 08 (17.4%)	30 (73.2%) 11 (26.8%)	0.288
Ethnic group						
White Not white	47 (94.0%) 03 (6.0%)	33 (89.2%) 04 (10.8%)	0.452	44 (95.7%) 02 (4.3%)	36 (87.8%) 05 (12.2%)	0.247
Age						
≤ 59 years > 59 years	28 (56.0%) 22 (44.0%)	17 (45.9%) 20 (54.1%)	0.354	25 (54.3%) 21 (45.7%)	20 (48.8%) 21 (51.2%)	0.604
Tobacco^#^						
No Yes	04 (8.5%) 43 (91.5%)	05 (14.7%) 29 (85.3%)	0.481	05 (11.6%) 38 (88.450	04 (10.5%) 34 (89.5%)	1.000
Alcohol^#^						
No Yes	08 (17.0%) 39 (83.0%)	07 (20.6%) 27 (79.4%)	0.683	08 (18.6%) 35 (81.4%)	07 (18.4%) 31 (81.6%)	0.983
Tumor site						
Tongue Floor of mouth	34 (68.0%) 16 (32.0%)	26 (70.3%) 11 (29.7%)	0.821	31 (67.4%) 15 (32.6%)	29 (70.7%) 12 (29.3%)	0.737
T stage						
T1 T2	19 (38.0%) 31 (62.0%)	09 (24.3%) 28 (75.7%)	0.177	18 (39.1%) 28 (60.9%)	10 (24.4%) 31 (75.6%)	0.142
Local recurrence						
No Yes	41 (82.0%) 09 (18.0%)	31 (83.8%) 06 (16.2%)	0.828	37 (80.4%) 09 (19.6%)	35 (85.4%) 06 (14.6%)	0.543
Regional recurrence						
No Yes	45 (90.0%) 05 (10.0%)	28 (75.7%) 09 (24.3%)	0.072	42 (91.3%) 04 (8.7%)	31 (75.6%) 10 (24.4%)	**0.047**
Lymphatic embolization						
No Yes	38 (76.0%) 12 (24.0%)	24 (64.9%) 13 (35.1%)	0.257	36 (78.3%) 10 (21.75)	26 (63.4%) 15 (36.6%)	0.127
Blood embolization						
No Yes	43 (86.0%) 07 (14.0%)	32 (86.5%) 05 (13.5%)	0.948	39 (84.8%) 07 (15.2%)	36 (87.8%) 05 (12.2%)	0.683
Perineural infiltration						
No Yes	26 (52.0%) 24 (48.0%)	17 (45.9%) 20 (54.1%)	0.577	25 (54.3%) 21 (45.7%)	18 (43.9%) 23 (56.1%)	0.331
Muscular infiltration						
No Yes	11 (22.0%) 39 (780%)	05 (13.5%) 32 (86.5%)	0.312	10 (21.7%) 36 (78.3%)	06 (14.6%) 35 (85.4%)	0.393
Salivary gland infiltration						
No Yes	34 (68.0%) 16 (32.0%)	24 (64.9%) 13 (35.1%)	0.759	33 (71.7%) 13 (28.3%)	25 (61.0%) 16 (39.0%)	0.288
Lymph-nodal status^#^						
pN0 pN +	32 (84.2%) 06 (15.8%)	18 (69.2%) 08 (30.8%)	0.155	29 (82.9%) 06 (17.1%)	21 (72.4%) 08 (27.6%)	0.314
Malignancy grading						
More differentiated Less differentiated	38 (76.0%) 12 (24.0%)	31 (83.8%) 06 (16.2%)	0.376	34 (73.9%) 12 (26.1%)	35 (85.4%) 06 (14.6%)	0.188

^#^Excluding patients with lost records

#### Hot spot evaluation

The mean value of ILVD was 45.90 lymphatic vessels/mm^2^. For statistical analyses, OSCCs were classified as having low ILVD (< 46 vessels/mm^2^) or high ILVD (≥ 46 vessels/mm^2^). ILVD exhibited no association with clinicopathologic or demographic parameters. In contrast, the majority of patients who had regional recurrence had a high ILVD (p = 0.047), as showed in Table 2*.* ILVD exhibited no significant association with histopathological malignancy grading. However, there was a tendency for patients with higher occurrence of metastasis to present a high ILVD (Table 2).

### Survival analysis

The clinical follow-up for the 87 patients with OSCC ranged from 5.4 to 272.1 months (mean 82.2 ± 63.1) [22]. At the end of the follow-up period, 33 patients (38%) were alive and disease-free, 19 patients (22%) had died of recurrence (local, regional or distant), 28 patients (32%) had died from causes other than tumor, and 7 (8%) were considered lost to follow-up because they reached a clinical outcome in less than 5 years.

**Fig. 2:**
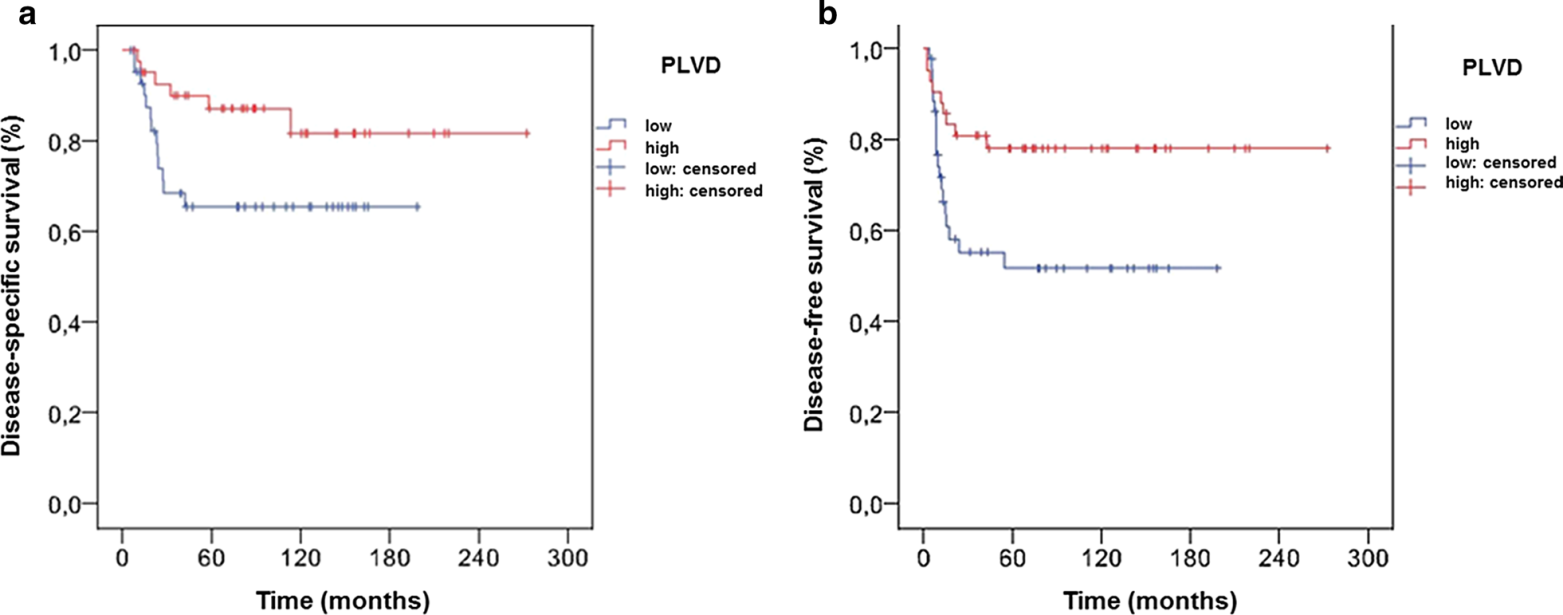
Disease-specific (**a**) and disease-free (**b**) survivals in oral squamous cell carcinoma patients, based on the peritumoral lymph vessel density (PLVD)

PLVD had no statistically significant influence on overall survival (sequential evaluation). However, a statistically significant correlation was observed between PLVD and disease-specific survival (*p* = 0.044) and disease-free survival (*p* = 0.016), as shown in Fig. 2. No association was observed between PLVD (hot spot) and overall, disease-specific or disease-free survival. There was no association among ILVD (sequential and hot spot) and overall, disease-specific or disease-free survival.

## Discussion

One of the fundamental questions in the management of patients with oral cancer consists in the treatment of the neck without the clinical presence of regional metastasis [24]. If metastasis is present, there is a consensus that the neck should be treated. However, in the clinical absence of metastasis, the decision is less clear [25, 26]. As a result of the limitation of accurate N staging, many surgeons opt to treat the neck of N0 patients electively, based mainly on size and location of the primary tumor, given that the prevalence of occult metastases in cervical lymph nodes of patients with head and neck SCCs exceeds 20% [8, 9]. Nevertheless, many N0 patients submitted to elective cervical dissection are treated unnecessarily and suffer with the morbidity caused by this surgical procedure [24].

Currently, the treatment selection of patients with head and neck SCC is based on the TNM clinical staging system [5] and this, in turn, is based on clinical parameters. However, the behavior of the tumor can differ widely within these TNM categories. Morphometric characteristics or molecular markers able to reflect the behavior of the primary tumor and the surrounding stroma can yield information about the metastatic potential of the tumor, allowing pathologists and clinicians to estimate the risk of lymph nodal metastasis in each patient, regardless the tumor size. These advances may empower the choice of the adequate therapeutic modality and limit the elective treatment of the neck to patients with higher risk of developing cervical metastases [26].

The association of LVD with regional metastases in OSCC remains matter of controversy. While most studies have reported that higher LVD is associated with local metastasis [12–15, 17, 18] and poorer survival [11, 18], more recent investigations confront these findings. Dedhia et al. [19] and Yan et al. [20] found that lower LVD was associated with cervical metastasis. The main limitation of these studies is the inclusion of patients in early as well as in advanced stages of the tumor. Thus, important differences might be masked by heterogeneity of the groups. To overcome this flaw, we restricted our analysis to patients with early OSCC.

Curiously, we found that PLVD (sequential or by hot spot) exhibited no statistically significant association with clinical evolution of the patients. However, patients with tumors that had high PLVD showed better outcome. Similarly, high PLVD (hot spot) was associated with lower occurrence of perineural infiltration in the OSCCs assessed. Our findings differ from the results of the studies cited above, that included patients in all clinical stages. Higher LVD has been also found to be associated with overexpression of VEGF-C, VEGF-D, and HIF-1α [17, 27], markers of local aggressiveness of the tumor. On the other hand, our results corroborated those of Dedhia et al. [19], and Yan et al. [20], who showed longer survival for patients whose tumors exhibited high PLVD (sequential). All these conflicting findings show how complex is the biology of OSCC.

The intriguing finding of the association between high PLVD and better prognosis for OSCCs demands further investigation. One may hypothesize that in early-stage OSCC, the peritumoral lymph vessels might have a protective role rather than be involved in the metastatic process. The lymphatic system is responsible for the immune function, tissue fluid homeostasis and the absorption of dietary fat [28]. As the inflammation has been shown to exert a dual role in the neoplastic development, the peritumoral lymph vessels in early-stage OSCC may be part of a—mostly unsuccessful—attempt of anti-tumoral response of the host. Indeed, the expression of VEGF-C has been shown to promote immune tolerance in murine melanoma [29]. On the other hand, the same group has showed that lymphangiogenesis promoted T cell infiltration and potentiated immunotherapy in murine melanoma [30]. Furthermore, Schoppmann et al. [31] suggested that tissue macrophages support the growth of the lymphatic network in the peritumoral region by expressing lymphangiogenic growth factors, such as VEGF-C. Peritumoral lymphatic vessels facilitate the recruitment of antigen-presenting cells, such as dendritic cells, the main recruiters of cytotoxic T cells in lymph nodes, the main defense mechanism of the host against tumor cells [32].

In agreement with the studies of Muñoz-Guerra et al. [11] and Zhao et al. [18], we found higher rate of regional recurrence in patients whose tumors had high ILVD. Despite not finding statistically significant association, most patients with regional invasion (pN +) in the present study also had high ILVD, corroborating other studies [12, 18, 27]. Miyahara et al. [15] and Sugiura et al. [17], although not making a distinction between PLVD and ILVD, also found greater regional invasion in patients with OSCC whose tumors had higher lymphatic density (Additional file 1: Supplementary Table 1).

Despite the advances in the knowledge on the mechanism of lymph nodal metastasis, many aspects of the intricate tumor biology remain unsolved. The exact function of the peri- and intra-tumoral lymph vessels, neoplastic lymphangiogenesis, and growth factors involved in these processes is still a mystery. Here, we have showed that the PLVD in early-OSCC may have a protective role rather than participate in the metastatic process, as traditionally reported. However, additional studies are necessary to confirm our results and to define precise predictive factors for the occurrence of occult regional metastases in OSCCs, in an effort to reduce the morbidity resulting from elective cervical dissection in many patients that exhibit pN0 on post-operative histopathologic exams.

## Supplementary information

**Additional file 1**. Studies about lymphatic vessel density significance in oral squamous cell carcinomas.

## Data Availability

The datasets used and/or analyzed during the current study are available from the corresponding author on reasonable request.

## Abbreviations

HE: Hematoxylin-eosin
ILVD: Intratumoral lymphatic vessel density
LVD: lymphatic vessel density
OSCC: Oral squamous cell carcinoma
PLVD: Peritumoral lymphatic vessel density
UICC: International Union Against Cancer
OSCC: Oral squamous cell carcinoma

## References

[1] Cao Y (2005). Opinion: emerging mechanisms of tumour lymphangiogenesis and lymphatic metastasis. Nat Rev Cancer.

[2] Tobler NE, Detmar M (2006). Tumor and lymph node lymphangiogenesis-impact on cancer metastasis. J Leukoc Biol.

[3] Achen MG, Stacker SA (2006). Tumor lymphangiogenesis and metastatic spread-new players begin to emerge. Int J Cancer.

[4] Das S and Skobe M. Lymphatic vessel activation in cancer. Ann N Y Acad Sci. 2008;021. 10.1196/annals.1413.021 18519976

[5] Sobin LHGM, Wittekind CH (2011). TNM: classification of malignant tumors.

[6] Almahmoudi R, Kasanen M, Sievilainen M (2019). Prognostic value of blood and lymphatic vessel markers in tongue cancer: a systematic review. Cancer Sci.

[7] Po Wing Yuen A, Lam KY, Lam LK (2002). Prognostic factors of clinically stage I and II oral tongue carcinoma-A comparative study of stage, thickness, shape, growth pattern, invasive front malignancy grading, Martinez-Gimeno score, and pathologic features. Head Neck.

[8] Byers RM, El-Naggar AK, Lee YY (1998). Can we detect or predict the presence of occult nodal metastases in patients with squamous carcinoma of the oral tongue?. Head Neck.

[9] Pimenta Amaral TM, Da Silva Freire AR, Carvalho AL (2004). Predictive factors of occult metastasis and prognosis of clinical stages I and II squamous cell carcinoma of the tongue and floor of the mouth. Oral Oncol.

[10] Okamoto M, Nishimine M, Kishi M (2002). Prediction of delayed neck metastasis in patients with stage I/II squamous cell carcinoma of the tongue. J Oral Pathol Med.

[11] Munoz-Guerra MF, Marazuela EG, Martin-Villar E (2004). Prognostic significance of intratumoral lymphangiogenesis in squamous cell carcinoma of the oral cavity. Cancer.

[12] Chung MK, Min JY, So YK (2010). Correlation between lymphatic vessel density and regional metastasis in squamous cell carcinoma of the tongue. Head Neck.

[13] Franchi A, Gallo O, Massi D (2004). Tumor lymphangiogenesis in head and neck squamous cell carcinoma: a morphometric study with clinical correlations. Cancer.

[14] Mafra RP, Serpa MS, Lima KC (2018). Immunohistochemical analysis of lymphatic vessel density and mast cells in oral tongue squamous cell carcinoma. J Craniomaxillofac Surg.

[15] Miyahara M, Tanuma J, Sugihara K (2007). Tumor lymphangiogenesis correlates with lymph node metastasis and clinicopathologic parameters in oral squamous cell carcinoma. Cancer.

[16] Siriwardena BS, Kudo Y, Ogawa I (2008). VEGF-C is associated with lymphatic status and invasion in oral cancer. J Clin Pathol.

[17] Sugiura T, Inoue Y, Matsuki R (2009). VEGF-C and VEGF-D expression is correlated with lymphatic vessel density and lymph node metastasis in oral squamous cell carcinoma: implications for use as a prognostic marker. Int J Oncol.

[18] Zhao D, Pan J, Li XQ (2008). Intratumoral lymphangiogenesis in oral squamous cell carcinoma and its clinicopathological significance. J Oral Pathol Med.

[19] Dedhia A, Gosavi S, Sharma B (2018). Low lymphatic vessel density correlates with lymph node metastasis in oral squamous cell carcinoma. J Dent (Shiraz).

[20] Yan J, Jiang Y, Ye M (2014). The clinical value of lymphatic vessel density, intercellular adhesion molecule 1 and vascular cell adhesion molecule 1 expression in patients with oral tongue squamous cell carcinoma. J Cancer Res Ther.

[21] de Sousa EA, Lourenco SV, de Moraes FP (2015). Head and neck squamous cell carcinoma lymphatic spread and survival: Relevance of vascular endothelial growth factor family for tumor evaluation. Head Neck.

[22] Faustino SE, Oliveira DT, Nonogaki S (2008). Expression of vascular endothelial growth factor-C does not predict occult lymph-node metastasis in early oral squamous cell carcinoma. Int J Oral Maxillofac Surg.

[23] Bryne M, Koppang HS, Lilleng R (1989). New malignancy grading is a better prognostic indicator than Broders' grading in oral squamous cell carcinomas. J Oral Pathol Med.

[24] Kowalski LP, Sanabria A (2007). Elective neck dissection in oral carcinoma: a critical review of the evidence. Acta Otorhinolaryngol Ital.

[25] Warburton G, Nikitakis NG, Roberson P (2007). Histopathological and lymphangiogenic parameters in relation to lymph node metastasis in early stage oral squamous cell carcinoma. J Oral Maxillofac Surg.

[26] Takes RP, Rinaldo A, Rodrigo JP (2008). Can biomarkers play a role in the decision about treatment of the clinically negative neck in patients with head and neck cancer?. Head Neck.

[27] Liang X, Yang D, Hu J (2008). Hypoxia inducible factor-alpha expression correlates with vascular endothelial growth factor-C expression and lymphangiogenesis/angiogenesis in oral squamous cell carcinoma. Anticancer Res.

[28] Stacker SA, Williams SP, Karnezis T (2014). Lymphangiogenesis and lymphatic vessel remodelling in cancer. Nat Rev Cancer.

[29] Lund AW, Duraes FV, Hirosue S (2012). VEGF-C promotes immune tolerance in B16 melanomas and cross-presentation of tumor antigen by lymph node lymphatics. Cell Rep.

[30] Fankhauser M, Broggi MAS, Potin L, et al. Tumor lymphangiogenesis promotes T cell infiltration and potentiates immunotherapy in melanoma. Sci Transl Med. 2017;9. 10.1126/scitranslmed.aal4712 28904226

[31] Schoppmann SF, Birner P, Stockl J (2002). Tumor-associated macrophages express lymphatic endothelial growth factors and are related to peritumoral lymphangiogenesis. Am J Pathol.

[32] Maula SM, Luukkaa M, Grenman R (2003). Intratumoral lymphatics are essential for the metastatic spread and prognosis in squamous cell carcinomas of the head and neck region. Can Res.

