# Supplementary material for: Association of lymph vessel density with occult lymph node metastasis and prognosis in oral squamous cell carcinoma
Source: BMC Oral Health. 2021 Mar 11;21:114. doi: 10.1186/s12903-021-01459-6 (PMC7948342; doi:10.1186/s12903-021-01459-6)
Supplement: Supplementary file 1 — Additional file 1. Studies about lymphatic vessel density significance in oral squamous cell carcinomas. [file 12903_2021_1459_MOESM1_ESM.docx]

**Association of lymph vessel density with occult lymph node metastasis and prognosis in oral squamous cell carcinoma**

Simone E. S. Faustino^1^ Kellen C. Tjioe^1,2^, Agnes Assao^1^, Michele C. Pereira^3^, André L. Carvalho^4^, Luiz P. Kowalski^5^ and Denise T. Oliveira^1^

^1^ Department of Surgery, Stomatology, Pathology, and Radiology, Area of Pathology, Bauru School of Dentistry, University of São Paulo, Bauru, São Paulo, Brazil;

^2^ Oral Oncology Center, Aracatuba School of Dentistry, São Paulo State University (Unesp), Aracatuba, São Paulo, Brazil;

^3^ Federal University of São João Del Rei – Midwest *Campus* Dona Lindu, Divinópolis, Minas Gerais, Brazil;

^4^ Department of Head and Neck Surgery, Barretos Cancer Hospital, Barretos, São Paulo, Brazil;

^5^ Department of Head and Neck Surgery and Otorhinolaryngology, A.C. Camargo Cancer Hospital, São Paulo, São Paulo, Brazil.

Supplementary table 1 – Characteristics of the studies about lymphatic vessel density significance in oral squamous cell carcinomas.

| **Authors (Year)** | **N** | **Tumor location** | **Stage of tumor** | **Primary Antibody** | **Results** |
| --- | --- | --- | --- | --- | --- |
| Franchi et al. (2004) | 18 | Oral cavity* | I-IV | D2-40 | - Lymphatic vessels more numerous and larger in the peritumoral area compared with within the tumor. - High peritumoral lymphangiogenesis associated with increased risk of lymph node metastasis |
| Muñoz-Guerra et al. (2004) | 61 | 35=Tongue  26=FOM | I-II | PA2.26 | - Intratumoral lymphangiogenesis (IL) associated with locoregional recurrence - Significant association between IL and disease-free survival |
| Miyahara et al. (2007) | 110 | 46=Tongue  64=Other oral region | I-IV | D2-40 | - LVD is correlated with lymph node metastasis and lower survival rates. |
| Ohno et al. (2007) | 50 | 38=Tongue  8=FOM  2=Gingiva  2=Buccal mucosa | I-IV | D2-40 | IL varies depending on the area of the tumor (higher in superficial peritumoral and intratumoral areas than in deep peritumoral and intratumoral areas). |
| Siriwardena et al. (2007) | 54 | Oral cavity* | I-IV | D2-40 | - Number of lymph vessels is higher in tumors with lymph node metastasis. - VEGF-C expression is associated with lymphatic invasion, lymph node metastasis, and number of lymph vessels. |
| Liang et al. (2008) | 65 | 41=Tongue  24=Buccal mucosa | I-IV | LYVE-1 | LVD is associated to the overexpression of VEGF-C and HIF-1α. |
| Zhao et al. (2008) | 86 | 39=Tongue  13=FOM  22=Buccal mucosa  12=Gingiva | I-IV | D2-40 | - Higher intratumoral lymphatic density (ILD) and peritumoral lymphatic density (PLD) are associated with the presence of lymph node metastasis at the time of diagnosis. - Higher ILD was significantly associated with a higher incidence of intratumoral lymphatic invasion, peritumoral lymphatic invasion and recurrence of tumor. - Patients with higher ILD exhibited shorter 5-year cumulative and disease-free survival. |
| Chung et al. (2009) | 62 | Tongue | I-IV | gp36 | - ILD is more predominant than PLD. - ILD is strongly correlated with regional metastasis. |
| Sugiura et al. (2009) | 160 | 65=Tongue  64=Gingiva  18=Buccal mucosa  12=FOM  1=Lip | I-IV | D2-40 | LVD is associated with lymph node metastasis, and increased VEGF-C and VEGF-D expressions. |
| Yan et al. (2014) | 80 | Tongue | I-IV | D2-40 | - LVD is higher in normal vs tumoral tissue. - Low LVD is associated with cervical lymph node metastasis. |
| Dedhia et al. (2018) | 50 | Not specified | I-IV | D2-40 | - LVD is higher in tumoral vs normal tissue. - Low LVD is associated with lymph node metastasis. |
| Mafra et al. (2018) | 56 | Tongue | I-IV | D2-40 | Higher ILD is associated with advanced clinical stages and lymph node metastasis. |

*Oral cavity: Specific anatomic site nor specified; FOM: Floor of the mouth; IL: Intratumoral lymphangiogenesis; ILD: intratumoral lymphatic density; PLD: peritumoral lymphatic density; LVD: lymphatic vessel density.
